# Lower pollen nutritional quality delays nest building and egg laying in *Bombus terrestris audax* micro-colonies leading to reduced biomass gain

**DOI:** 10.1007/s13592-021-00885-3

**Published:** 2021-09-27

**Authors:** Jordan T. Ryder, Andrew Cherrill, Helen M. Thompson, Keith F. A. Walters

**Affiliations:** 1 Centre for Integrated Pest Management, Harper Adams University, Newport, Shropshire, TF10 8NB UK; 2 Syngenta, Jealott’s Hill, Berkshire Bracknell, RG42 6EY UK; 3 Division of Ecology and Evolution, Imperial College London, Silwood Park Campus, Berkshire Ascot, SL5 7PY UK; 4 Department of Biological Sciences, University of Chester, Parkgate Road, Cheshire Chester, CH1 4BJ UK

**Keywords:** *Bombus terrestris audax*, Nutrition, Amino acid profile, Pollen mixing, Colony performance

## Abstract

The performance of *Bombus terrestris* micro-colonies fed five diets differing in pollen species composition and level of nine essential amino acids (EAA; leucine, lysine, valine, arginine, isoleucine, phenylalanine, threonine, histidine, methionine) was assessed for 37 days by recording total biomass gain, nest building initiation, brood production (eggs, small and large larvae, pupae, drones), nectar, and pollen collection. Stronger colony performance was linked to higher amino acid levels but no consistent differences in biomass gain were recorded between mono- and poly-species diets. Poorest performance occurred in micro-colonies offered pure oilseed rape (OSR) pollen which contained the lowest EAA levels. Reduced micro-colony development (delayed nest initiation and lower brood production) was related to OSR proportion in the diet and lower EAA levels. Results are discussed in relation to selection of plant species in the design of habitats to promote bee populations.

## Introduction

Bumblebees (*Bombus* spp.) are a key group of highly efficient wild pollinators, which forage on a wide variety of flowers and plants (Reynolds and Fenster 2008). Foraging behaviour of individual workers is highly selective (Harmon-Threatt et al. 2017), and it has been proposed that the nutritional content of pollen affects flower selection (Nicolson 2011). Individual foragers of *Bombus terrestris* maintain a degree of floral consistency (Goulson 2009) whilst differences in preferences between foragers result in the utilisation of a wider range of plant species at the colony level (Free 1970). The resultant poly-floral larval diets have been reported to strengthen colony development (Baloglu and Gurel 2015).

The importance of pollen diversity is widely recognised, and habitat management schemes have focused on increasing floral diversity to enhance pollinator populations (Carvell et al. 2006). In the UK, environmental stewardship schemes promote a range of species mixes and sowing options to enhance botanical diversity of arable landscapes (e.g. Carvell et al. 2007), but further work is required to optimise their impact on wild bee colonies (Albrecht et al. 2007).

The ratio and level of the major nutritional components of pollen, including proteins and their constituent amino acids, lipids (including phytosterols), carbohydrates, and vitamins and secondary metabolites such as carotenoids and flavonoids, are related to its nutritional value for bumblebees (Vaudo et al. 2020), and the degree to which pollen meets the larval requirements varies between plant species (Filipiak 2019; Vanderplanck et al. 2014; Somme et al. 2015). Bumblebees have been shown to favour more protein-rich pollens (Leonhardt and Blüthgen 2012; Kitaoka and Nieh 2009) but amino acid composition is a better determinant of pollen quality for bees than total protein content (Nicolson, 2011; Moerman et al. 2015, 2017; Stabler et al. 2015).

Assessing the role of selective foraging on colony success relies on an understanding of the effect of pollen diet on bumblebee colony performance. Colony development and brood production, bee physiology, and immune system function have all been studied (Dance et al. 2017), with colony fitness being assessed using parameters such as egg production, larval weight, larval ejection, adult body size, adult longevity, and the number of active foragers (Tasei and Aupinel 2008a; Kitaoka and Nieh 2009; Kriesell et al.^ 2017^; Vanderplanck et al. 2014). Colony responses to defined pollen diets may be investigated using laboratory-based micro-colony experiments to identify response parameters that can subsequently be verified in queenright colonies (Génissel et al. 2002; Tasei and Aupinel 2008b). Micro-colony studies suggest that mixed-species pollen diets are more favourable than mono-species diets (Génissel et al. 2002; Vanderplanck et al. 2014). As amino acid content of pollen is thought to be a primary driver of bumblebee colony success (Moerman et al. 2015, 2017; Stabler et al. 2015), pollen diversity may increase the potential for both essential amino acids and other essential nutritional components being included. Studies in bumblebees are limited, however, and further work comparing nutritional content of pollen diets with colony performance are required to identify specific biological mechanisms leading to colony level outcomes.

This study investigates the effect of five defined pollen diets on the development of *B. terrestris audax* micro-colonies to test the hypothesis that colony performance is defined, in part, by their amino acid profiles and the diversity of pollen species included.

## Materials and methods

Queenless *B. terrestris audax* micro-colonies were established using worker bees from stock colonies obtained from Agralan Ltd., Swindon, UK (originating from Biobest®, Belgium). Prior to use, colonies were fed ad libitum on Biobest standard pollen mix and proprietary liquid sugar solution and maintained for a 7-day acclimation period in a CE room at 27 °C, 65% RH, with an 8:16 light–dark cycle (Elston et al. 2013).

Micro-colony arenas (modified from Elston et al. 2013) consisted of 500-ml open-topped plastic containers (11 cm diameter × 7 cm deep), closed with muslin mesh. The base of each arena was lined with filter paper, and a small ball of cotton wool was added to encourage nest building.

Artificial nectar solution (60%, w/v Rowse Pure Honey and water) was offered ad libitum to micro-colonies in lidded plastic feeding tubes (length = 10 cm, Φ = 1 cm, with an upward-facing feeding hole (Φ = 2 mm) pierced at one end) inserted at a 30° angle through a hole in the side of the colony cages. Pollen was offered using a similar tube but with a 10 × 20-mm feeding trough, inserted horizontally at 180° in each direction from the nectar tube.

Both feeding tubes were weighed and replaced with new pre-weighed tubes containing fresh pollen at 2-day intervals ensuring that a minimum of 2 g of pollen and 8 ml nectar were available throughout the experiment. Three worker bees were transferred from stock colonies to each micro-colony cage at the start of the experiment; bees that remained inactive for 1 h after transfer were replaced.

### Treatments

One of five commercially sourced dried pollen species or pollen mixes were offered to micro-colonies, selected to allow comparison of diets containing single pollen species with those with diverse pollen species. By combining different pollen sources, a series of three diets containing varying proportions of oilseed rape pollen were also produced (“graded mixes”; see below). The pollen mix used both as a control and as a base for the preparation of graded mixes was an industry standard used to rear commercially marketed colonies of *B. terrestris* used for pollination of glasshouse crops, thus thought to provide all nutritive requirements. Commercially procured pollens used in treatments were reported to be collected directly from plants, although palynological analysis of the organic chestnut pollen highlighted the presence of other species:

Controls: Biobest standard pollen mix (from Biobest®, Belgium; “standard pollen mix”), *n* = 18

Experimental treatments:

T1—organic chestnut pollen mix (TOCA®, Spain; *Castanea sativa*; “chestnut pollen mix”), *n* = 15

T2—Pure *Camellia* pollen (Simianshan®, China; *Camellia* spp.”; “Camellia”), *n* = 15

T3—Pure oilseed rape pollen (Simianshan®; China; *Brassica napus*; “OSR”), *n* = 15

T4—A 50%:50% mixture of Biobest standard pollen mix and pure OSR pollen (“standard pollen/OSR”), *n* = 16

All pollen treatments were homogenised using a wet and dry grinder (Andrew James Ltd., UK), and stored at –20 °C until used in the experiment. Replication varied slightly with between 15 and 18 micro-colonies per control or treatment, due to a limited availability of worker bees from the stock colony, but in all cases, stock colonies were established using 3 bees per micro-colony. The experiment was run under the conditions used during acclimation and was terminated after 37 days (by which point drone production had been recorded in all treatments).

#### Palynological analysis

Three sample slides were prepared from each treatment for pollen composition analysis, using the method of Moore et al. (1991). Pollen identification was carried out at 400 × magnification using a Microtec compound microscope (TEC Microscopes Ltd., UK). A minimum of 50 grains selected at random from each slide were identified to at least genus (Moore et al. 1991) and percentage contribution of each genus/species to the sample was determined. Pollen grains that could not be identified were recorded as “unknown”.

### Amino acid analysis

Three sub-samples (5–10 mg) of each of the homogenised pollen mixes from each treatment were analysed (Alta Bioscience, Birmingham) to determine amino acid content after acid hydrolysis of lypholised samples according to European Pharmacopoeia methodology (CoE 2017); this is an ISO 17,025:2005-accredited method with a limit of quantification of 5 nmol. This method reflects the total sum of amino acids (protein incorporated and free in solution), excluding tryptophan and cysteine/cystine (which are usually lost during acid hydrolysis) and creatine and creatinine (which cannot be analysed using this method). The results were presented in two groups, essential amino acids (de Groot 1953), which must be obtained from the diet, and non-essential amino acids, which can be supplemented by the diet. The data was also presented for each of the nine essential amino acids.

### Assessments

#### Mortality

Mortality of the founding worker bees (if any) in each micro-colony was recorded at the end of each 2-day period, and dead bees were removed but not replaced. Worker bees and drones were distinguished by the presence or absence of a sting (Goulson, 2009).

#### Nectar and pollen collection

Collection of nectar and pollen by each micro-colony was calculated from the difference between feeder weight at the start and end of each 2-day period and expressed as mean collection (g) per bee/2 days (taking account of recorded mortality).

#### Nest building

Each micro-colony was observed at the end of each 2-day assessment period and the first nest building activity (nest initiation: defined as either wax cell or honey pot construction) recorded.

#### Final micro-colony performance

After the 37-day experimental period, micro-colonies were euthanized by freezing at –20 °C for 24 h. Nests (including all the wax material and brood inside) were weighed and dissected, and the number of eggs, small larvae (< 0.8 cm across when curled), large larvae, and pupae were recorded. The number of drones produced were counted and weighed. The sum of the drone weight and nest weight (including immature bees) was recorded as “colony biomass gain”.

### Statistical analysis

Statistical analysis was conducted using R version 4.0.2 (R Core Team, 2017), with packages “MASS” (Venables and Ripley 2002), “lmtest” (Zeileis and Hothorn 2002), “multcomp” (Hothorn et al. 2008), “multcompView” (Graves et al. 2019), and “emmeans” (Length 2020). All data for parametric tests were checked for normality and Log or sqrt transformations were applied where necessary. Factor reduction was conducted following normal conventions of step-wise deletion starting with the fitting of the maximal model with all explanatory variables and interactions. This allowed for the removal of non-significant interaction terms and variables. Where appropriate, explanatory variable levels that did not differ significantly from each other were combined into a single factor for analysis. Each step of factor reduction was checked with an ANOVA between models in order to ensure the model explanatory power was not statistically affected, thus allowing construction of the minimum adequate model for all statistical tests conducted (Crawley 2013).

#### Pollen amino acid composition

Prior to analysis, a square root transformation was applied to normalise data for total amino acid (TAA) content, total non-essential amino acids (non-EAA), and total essential amino acids (EAA) of each pollen treatment (g/100 g) which were each subjected to an ANOVA. Tukey post hoc tests were used to confirm where significant differences occurred between treatments.

#### Nectar and pollen collection

Data on collection of nectar and pollen were subjected to square root and log transformations respectively to meet assumptions of normality. The effects of treatments on nectar and pollen collection over time were analysed using repeated measures ANOVA, with micro-colony replication identity (ID) added as error within the model. Tukey post hoc tests were used to confirm where significant differences occurred.

#### Nest initiation

Time of nest initiation (first nest building activity) was treated as a binomial response variable. In order to compare between pollen treatments against time, a generalised linear model (GLM) with binomial error structure was utlised.

#### Worker mortality and brood production data

The number of dead workers, or the number of eggs, larvae (both early and late instars), pupae, and drones present at the end of the experiment were compared between treatments using GLM with Poisson error distribution, and quasi-Poisson error distribution where data was over-dispersed.

#### Colony biomass gain

The weight gain of the micro-colonies in each treatment at the end of the experimental period was analysed using a one-way ANOVA. Turkey’s post hoc test was used to confirm where significant differences occurred.

## Results

### Palynological analysis

Sweet chestnut pollen (*Castanea sativa*) represented 65.5% of the grains identified from pollen marketed as “organic chestnut pollen”, with the remainder dominated by *Prunus* spp., *Lotus corniculatus* and *B. napus* (Table I). These species also constituted 51.2% of the commercially sourced standard pollen mix (used as control) which included eight different genera. Camellia and OSR treatments were found to be pure.

**Table I:** Palynological analysis of the commercially sourced pollens used in the experimental treatments. The percentage of pollen grains for each species is a mean of three samples

Commercial name	Pollen species	% pollen grains
Standard pollen mix	*Brassica napus*	27.6
	*Salix* spp.	18.8
	*Taraxacum officinale*	16.3
	*Prunus* spp.	14.2
	*Ranunculus repens*	8.1
	*Pinus* spp.	5.6
	*Castanea sativa*	5.2
	*Lotus corniculatus*	4.2
Camellia	*Camellia* spp.	100.0
Chestnut pollen mix	*Castanea sativa*	65.5
	*Prunus* spp.	17.2
	*Lotus corniculatus*	9.5
	*Brassica napus*	7.0
	Unknown	0.8
Oilseed rape	*Brassica napus*	100.0
Standard pollen/OSR	*Brassica napus*	63.8
	*Salix* spp.	9.4
	*Taraxacum officinale*	8.1
	*Prunus* spp.	7.1
	*Ranunculus repens*	4.1
	*Pinus* spp.	2.8
	*Castanea sativa*	2.6
	*Lotus corniculatus*	2.1

### Pollen amino acid composition

#### Total amino acid content

**Figure 1.**
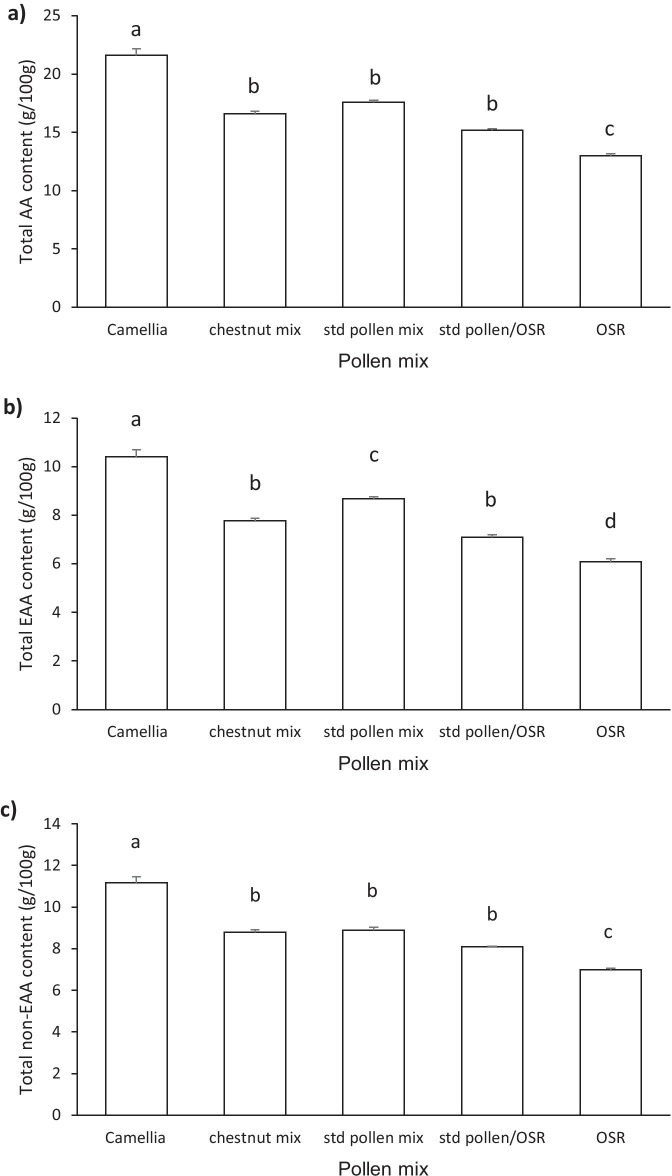
Amino acid content of the five pollen mixes (g/100 g) (OSR = oilseed rape). **a** Total amino acid (TAA). **b** Total essential amino acid (EAA). **c** Total non-essential amino acid (non-EAA) used in treatments. Mean (± S.E.) of three samples from each treatment. Bars with the same letter are not significantly different (*p* > 0.05).

There was a significant difference between TAA content of treatments (*F* = 126.3; d.f. = 4, 10; *p* < 0.001; Figure 1a). Tukey post hoc tests confirmed that TAA was higher in the Camellia pollen treatment than in all other treatments (*p* < 0.001). The OSR treatment had a significantly lower TAA content than all other treatments (*p* < 0.001), but no difference was recorded between the chestnut pollen mix, standard pollen mix, and standard pollen/OSR pollen mix (*p* > 0.05).

#### Total non-essential amino acid

A significant difference between the total NAA content of treatments was identified (*F* = 94.95; d.f. = 4, 10; *p* < 0.001) (Figure 1c). Camellia pollen had higher levels of NAA than all other treatments (*p* < 0.001), and OSR had lower levels than both the chestnut pollen mix and the standard pollen mix treatments (*p* < 0.001).

#### Total essential amino acids

Significant differences were also recorded between the total EAA content of the pollen treatments (*F* = 112.7; d.f. = 4, 10; *p* < 0.001; Figure 1b). Tukey post hoc tests confirmed that Camellia pollen had higher levels of EAA than all other treatments (*p* < 0.001), with OSR having lower levels than the other treatments (*p* < 0.001). The standard pollen mix had higher levels of EAA than the chestnut pollen mix (*p* < 0.05) and the standard pollen/OSR mix (*p* < 0.001).

#### Individual essential amino acids

There was a statistically significant interaction between treatment and the level of individual EAAs (*F* = 13.77; d.f. = 32, 90; *p* < 0.001). Tukey post hoc tests confirmed that significant differences in levels of individual EAAs occurred between treatments (Table II).

**Table II:** The essential amino acid content of the five pollen mixes used in the treatments and control. Figures represent mean amino acid content (g/100 g ± standard error). Within columns (amino acids), means with the same letters are not significantly different (*P* > 0.05)

	Leucine	Lysine	Valine	Arginine	Isoleucine	Phenylalanine	Threonine	Histidine	Methionine
Camellia	1.853 ± 0.056**a**	1.523 ± 0.043**a**	1.233 ± 0.038**a**	1.153 ± 0.041**a**	1.133 ± 0.033**a**	1.103 ± 0.033**a**	1.027 ± 0.029**a**	0.818 ± 0.020**a**	0.565 ± 0.010**a**
Chestnut pollen mix	1.330 ± 0.012**b**	1.127 ± 0.015**b**	0.928 ± 0.012**b**	0.896 ± 0.005**b**	0.844 ± 0.008**b,d**	0.799 ± 0.008**b,d**	0.756 ± 0.017**b**	0.717 ± 0.022**a**	0.381 ± 0.009**b,c**
Standard pollen mix	1.440 ± 0.036**b**	1.393 ± 0.029**a**	1.011 ± 0.015**b**	0.955 ± 0.004**b**	0.929 ± 0.009**b,d**	0.907 ± 0.010**b**	0.866 ± 0.025**a**	0.729 ± 0.009**a**	0.452 ± 0.003**a,c**
Standard pollen/OSR	1.193 ± 0.015**c**	0.991 ± 0.007**c**	0.855 ± 0.015**b**	0.787 ± 0.015**b,c**	0.750 ± 0.012**d**	0.739 ± 0.011**c,d**	0.727 ± 0.008**b**	0.700 ± 0.013**a,b**	0.358 ± 0.006**b,c**
OSR	1.005 ± 0.020**d**	0.878 ± 0.021**c**	0.753 ± 0.021**c**	0.715 ± 0.019**c**	0.640 ± 0.005**c**	0.620 ± 0.015**c**	0.606 ± 0.016**b**	0.573 ± 0.005**b**	0.294 ± 0.006**b**

Significant trends in the relative levels of different amino acids between pollen mixes (treatments) were identified as follows:

Higher levels of leucine, valine, arginine, isoleucine, and phenylalanine were recorded in Camellia pollen than in all other treatments (Table II). Continuing the trend, Camellia and the standard pollen mix had similar levels of lysine and threonine, and displayed higher levels than in all other treatments. Camellia and the standard pollen mix also had similar levels of methionine but higher levels than were found in OSR. Levels of histidine were similar in Camellia, the chestnut pollen mix, and the standard pollen mix, but these were again all higher than that in OSR.

Greater overlap in EAA levels were found between the chestnut pollen mix, standard pollen mix, and standard pollen/OSR mix, with no differences found for the levels of valine, arginine, isoleucine, histidine, and methionine. Chestnut and standard pollen mix also had similar levels of leucine and phenylalanine, but threonine and lysine were present at higher levels in the standard pollen mix. Chestnut pollen mix and the standard pollen/OSR mix had similar levels of valine, arginine, isoleucine, phenylalanine, threonine, histidine, and methionine, whilst chestnut pollen had higher levels of leucine and lysine.

The trend for OSR having lower levels of essential amino acids (Figure 1b; Table II) was reinforced by other findings. Levels of leucine, valine, and isoleucine were lower in the OSR pollen than those in all other treatments. In the other cases, no significant differences were found between pure OSR and standard pollen/OSR mix treatments for lysine, arginine, phenylalanine, threonine, or histidine, which again were the lowest recorded. For the remaining EAA investigated (methionine), levels were similar to those in both the chestnut pollen mix and the standard pollen/OSR mix again displaying the lowest levels of all treatments.

Comparing the three treatments containing different proportions of OSR pollen, the standard pollen treatments (with the lowest proportion) contained the highest levels of four of the EAAs investigated, with equal levels of valine, arginine, isoleucine, histidine, and methionine in the standard pollen and standard pollen/OSR mixes. In all cases, the standard pollen mix contained more of each EAA than the pure OSR treatment.

### Worker mortality

At the end of the 37-day experimental period, mortality of workers was low (6.3%). No differences in mortality were found between treatments (*z* =  −0.352; d.f = 1420, *p* > 0.05).

### Collection of honey solution

Repeated measures ANOVA showed that honey solution mean collection per bee varied with day (*F* = 14.55; d.f. = 1, 943; *p* < 0.001), but was not affected by treatment (*F* = 1.97; d.f. = 4, 74; *p* > 0.05).

### Pollen collection

Repeated measures ANOVA indicated that pollen collection per bee varied with both treatment (*F* = 12.11; d.f. = 4, 69; *p* < 0.001) and day (*F* = 15.55; d.f. = 1, 1085; *p* < 0.001). No interaction between treatment and day was found (*F* = 2.33; d.f. = 4, 1338; *p* < 0.05). A significantly higher weight of pollen was collected when bees were offered the standard pollen or chestnut pollen mixes than when the standard pollen/OSR, Camellia, or OSR pollen were available (Table III).

**Table III:** Post hoc *t*-test analysis of weight of pollen collected by micro-colonies offered different pollen mixes

*T*-test results				
	Standard pollen	Chestnut	Camellia	Oilseed rape
Chestnut pollen mix	*p* > 0.5	-	-	-
Camellia	***p* < 0.01**	***p* < 0.001**	-	-
Oilseed rape	***p* < 0.001**	***p* < 0.001**	*p* > 0.05	-
Standard mix/OSR	***p* < 0.001**	***p* < 0.001**	*p* > 0.05	*p* > 0.05

### Micro-colony biomass gain

**Figure 2.**
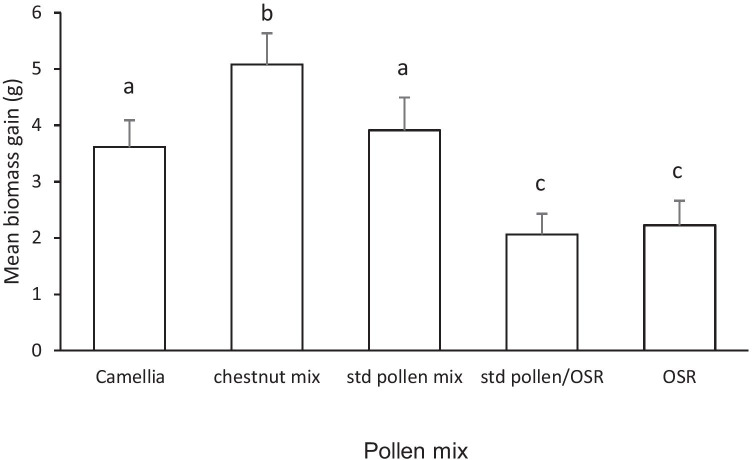
Mean (± S.E.) biomass gain (g) of micro-colonies exposed to treatments offered five pollen mixes (OSR = oilseed rape), over the 37 days of the experiment. Bars with the same letter are not significantly different (*p* > 0.05).

Micro-colony biomass gain over the 37 days of the experiment differed significantly between treatments (*F* = 5.81; d.f. = 4; 74, *p* < 0.001; Figure 2). Tukey post hoc analyses confirmed that lower colony biomass was recorded in the pure OSR and standard pollen/OSR mix treatments when compared to that recorded in the chestnut pollen treatment which attained the highest biomass gain (*p* < 0.01, *p* < 0.01 respectively). Biomass gains in the Camellia and standard pollen mix treatments were not significantly different and with a mean of 26% lower (*p* < 0.05) than that in the chestnut pollen mix, and 43% higher (*p* < 0.01) than those in the standard pollen/OSR mix or OSR treatments.

### Components of biomass gain

#### Initiation of nest building

**Figure 3.**
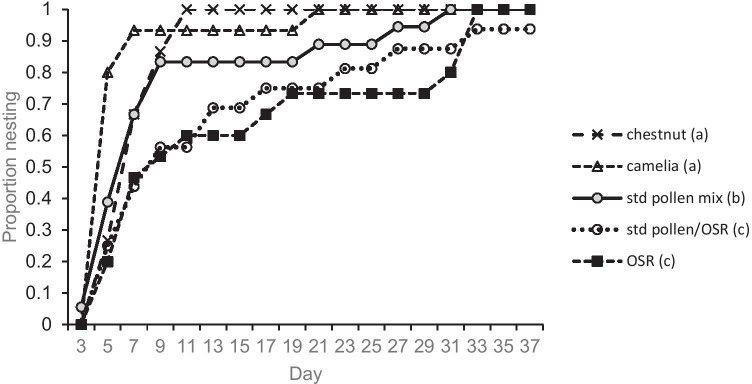
Cumulative proportion of micro-colonies displaying nest building activity on sequential assessment days, when offered five different pollen mixes (OSR = oilseed rape). Pollen sources with the same letter are not significantly different (*p* > 0.05).

The day on which nest building commenced in individual micro-colonies varied between treatments (Figure 3). During the creation of the minimum adequate model, no interaction between day and treatment was found and so “day” was removed from the model, although overall, a GLM with binomial error structure showed that the proportion of nesting micro-colonies increased with time (*z* = 14.95, d.f. = 1419, *p* < 0.001).

More than 90% of micro-colonies in the Camellia and chestnut pollen treatments had initiated nest building by days 7 and 11, respectively, with all having done so earlier (days 21 and 11) than in other treatments. Most (90%) micro-colonies in the standard pollen mix treatment had commenced nest building by day 27, with 100% having done so by day 31. All micro-colonies offered the pure OSR treatment which displayed nest building activity by day 33, but only 92% of micro-colonies in the standard pollen/OSR mix treatment had initiated nest building after 37 days (Figure 3).

The Camellia and chestnut treatments did not differ significantly and were combined into a single factor for analysis; these treatments had the earliest timing of nest initiation and thus formed the intercept for analysis.

The standard mix showed the second highest timing of initiation (*z* =  −3.19, d.f. = 1419, *p* < 0.01). The standard pollen/OSR mix and pure OSR pollen treatments did not differ, were combined, and displayed later nest initiation than all other treatments (*z* =  −8.29, d.f. = 1419, *p* < 0.001).

#### Brood production

For three factors, egg count, total larvae, and total small larvae, the standard pollen mix, chestnut mix, Camellia, and OSR treatments were found not to be significantly different; thus, following the standard approach, they were combined into a single factor for analysis during creation of the minimum adequate model. Analysis of the mature brood data found that standard pollen mix, chestnut mix, and Camellia treatments did not differ and were again combined into a single factor.

**Figure 4.**
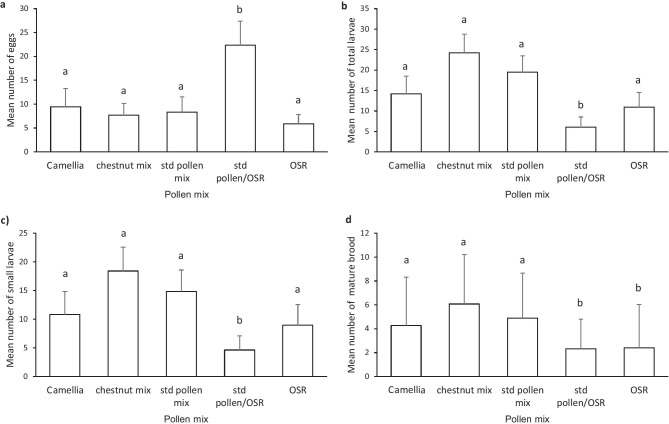
Mean (± S.E.) number of brood per micro-colony recorded (day 37 of the experiment) in treatments offered different commercially sourced pollen mixtures. Mean number of **a** eggs, **b** total larvae (small + large), **c** small larvae, and **d** older “brood” (including large larvae, pupae, and drones). Error bars show ± 1 standard error of the mean, calculated from linear models. Pollen source with the same letter are not significantly different (*p* > 0.05) .

Significantly more eggs were recorded in standard pollen/OSR mix (in which later nest initiation had also been recorded), than in all other treatments (Figure 4a; *t* = 3.64, d.f. = 77, *p* < 0.001).

The total number of larvae (small + large larvae) found in the micro-colonies was lower in the standard pollen/OSR mix treatment (Figure 4b; *t* =  −2.46, d.f. = 77, *p* < 0.05) than in the other treatments. In addition, significantly fewer small larvae were recorded in the standard pollen/OSR mix treatment than in other treatments (Figure 4c; *t* = 2.34, d.f. = 77, *p* < 0.05).

Significantly fewer older brood (total number of large larvae, pupae, and drones) were found in nests from both the standard pollen/OSR mix treatment (Figure 4d; *t* =  −2.70, d.f. = 76, *p* < 0.01) and the treatment fed pure OSR pollen (*t* =  −2.54, d.f. = 76, *p* < 0.05), the two treatments displaying the latest nest initiation.

## Discussion

Pollen represents the major protein source for brood of some pollinator species such as bumblebees (Roulston and Cane 2000). Larval diets containing diverse pollen species can favour bumblebee colony development (Génissel et al. 2002; Vanderplanck et al. 2014), and nutrient content (including components such as amino acids and lipids) may provide a mechanistic basis for this observation (Moerman et al. 2017; Vaudo et al. 2020). Kriesell et al. (2017) reported wide variation in amino acid content of pollen species recovered from individual pollen loads of foraging bumblebees, but lower variability in EAA content between loads, suggesting selective foraging may result in improved nutritional quality of diets fed to larvae. Establishment of requirements for important nutritional components such as amino acids will support selection, or directive breeding, of plants used in habitats designed to promote pollinators.

This study investigated the impact of nutritionally diverse pollen sources on performance of queenless *B. terrestris* micro-colonies; pollens utilised were devoid of morphological floral traits that would impact the results (Westerkamp and Claβen-Bockhoff 2007). Nest initiation and brood production were successful across treatments, and all pollen sources were utilised.

Low mortality of worker bees (6.3%) occurred across all treatments, honey solution collection did not differ between treatments, and both were similar to levels recorded in other studies (Elston et al. 2013), implying that all diets offered at least the minimum required nutrition for colony growth. Pollen collection, however, varied significantly with both time and treatment, possibly a response to nutrient content.

Micro-colonies offered the chestnut pollen mix (primarily 4 genera; 65.5% sweet chestnut) achieved the highest colony biomass gain, followed by pure Camellia pollen, and the standard pollen mix (8 genera; 27.6% OSR). The lowest biomass gains were recorded from micro-colonies fed either pure OSR pollen, or the standard pollen mix combined with OSR pollen (8 genera; 63.8% OSR). Thus, although the highest growth rate was associated with a diverse pollen source, no simple correlation between diverse pollen diets and biomass gain was identified. Instead, evidence was obtained that when diets contained high proportions of OSR pollen, there was a depression of biomass gain. Thus, species composition, as well as diversity, was important. In addition, there is clear evidence supporting the concept that although amino acids are an important component, distinguishing between the total nutritional value of different pollen species will require studies of other nutrient classes, such as lipids.

There were significant differences in the timing of nest initiation (and associated egg production) following establishment of micro-colonies. Colonies offered a diet of the chestnut pollen mix or pure *Camellia* pollen commenced nest building activities earlier than those offered the standard pollen mix. Micro-colonies offered higher proportions of OSR pollen took significantly longer to initiate nest building, possibly reflecting egg production in holometabolous insects being a nutrient-limited process (Wheeler, 1996; Hoover et al. 2006). Responsiveness of bumblebee queens initiating nests in spring is important as it can promote synchrony with periods of optimal floral resource availability (Geib et al. 2015); nest enlargement to accommodate eggs/larvae is undertaken by other castes (Michener 2007).

Brood recorded at the end of the experiment reflected similar responses to diet. Micro-colonies offered pure OSR produced fewer older brood. Significantly more eggs, but fewer larvae and older brood, were also recorded in the standard pollen/OSR mix treatment than in other treatments, suggesting that later nest building resulted in a later egg laying/hatch in these treatments. Consequently, micro-colonies offered pollen with a high proportion of OSR had fewer older brood (total number of large larvae, pupae, and drones) and lower colony biomass at the end of the experiment than those offered diets with lower levels of OSR pollen.

Previous micro-colony studies of the effect of nutrition on colony development rarely consider potential effects on nest building activity (Génissel et al. 2002). Many have terminated experiments earlier than in the current work (thus, data on later colony development were not collected) (Tasei and Aupinel 2008b) or encountered both oophagy and larval ejection, with associated difficulties when interpreting results (Génissel et al. 2002). This study indicates that assessment of colony success should not rely on the presence of larvae alone but in addition consider a range of other parameters. In this respect, total biomass gain may be a comprehensive parameter reflecting overall brood production or growth of the colony.

Carbohydrates, lipids, protein, vitamins, minerals, and starch have all been implicated as essential nutrients for honey bees but amino acid composition is most often used to assess nutritional quality (Cook et al. 2003). Lipids (in particular sterols) have been highlighted as important in brood production (Vanderplanck et al. 2014; Moerman et al. 2017; Vaudo et al. 2020), and bees appear to be able to regulate their lipid intake (Vaudo et al. 2016; Kraus et al. 2019). The suggestion that amino acid content of larval pollen resources is also a key factor determining bumblebee colony performance (Moerman et al. 2017) may offer a partial mechanism explaining the results obtained in this study.

Significant differences between treatments in the levels of nine of the amino acids reported as essential for honeybees (de Groot 1953) were recorded in the current study. The lowest level of each was found in the pure OSR pollen with significantly higher levels in pollens collected by the highest performing colonies (chestnut mix, Camellia, standard mix).

Cook et al. (2003) reported that honeybees preferentially foraged on oilseed rape compared to field bean (*Vicia faba*) pollen reflecting higher levels of valine, leucine, and isoleucine. Although bumblebee micro-colonies performed least well when fed on OSR pollen in the current study, it is notable that these three essential amino acids were present in lower quantities than in the other pollen diets investigated. In addition, when the three diets each containing different proportions of OSR pollen were offered to the bumblebee micro-colonies, those offered the diet containing the lowest proportion of OSR (thus the highest levels of the EAA) performed significantly better than those with the pure OSR pollen. This supports the suggestion that polylectic bees such as bumblebees may ameliorate the impact of nutritional deficiencies of some pollens by collecting from multiple species. Bumblebees frequently exploit flowers from several plant species in single foraging flights (Leonhardt and Blüthgen 2012; Kriesell et al.^ 2017^), and 2–8 species have been recorded in pollen loads taken from *Bombus lucorum* and *Bombus pascuorum* (Free 1970).

Previous studies suggest that species-rich habitats offer better resources than habitats containing lower floral diversity (Dance et al. 2017; Hass et al. 2018). This is thought to result from potential nutritional limitation of mono-species pollens, whereas poly-floral pollens may be nutritionally complimentary to each other (Moerman et al. 2015, 2017; Stabler, et al. 2015). Such theory has been widely accepted and diversity has been a key factor when creating and promoting pollinator-friendly land use, such as in environmental stewardship schemes. This study provides further data confirming the principle, and quantifying the impact on colony success using a wider range of colony characteristics than employed in most previous work, in combination with quantification of levels of total amino acids, total and individual EAA, and total non-EAA in dietary pollen. It was concluded that, in each case, colony performance was linked (in part) to amino acid content. The contention that nutritional deficiencies in individual pollen species could be ameliorated by selected poly-floral larval diets was supported. Future work should concentrate on analysis of key nutritional components of pollen, to support more informed selection of plant species for stewardship schemes designed to increase both polylectic and monolectic bumble bee species abundance (Carvell et al. 2007).

## Data Availability

The datasets generated and analysed during the current study are available from the corresponding author on reasonable request.

## Code availability

The code used for statistical analysis during the current study is available from the corresponding author on reasonable request.
